# Predicting self-harm within six months after initial presentation to youth mental health services: A machine learning study

**DOI:** 10.1371/journal.pone.0243467

**Published:** 2020-12-31

**Authors:** Frank Iorfino, Nicholas Ho, Joanne S. Carpenter, Shane P. Cross, Tracey A. Davenport, Daniel F. Hermens, Hannah Yee, Alissa Nichles, Natalia Zmicerevska, Adam Guastella, Elizabeth Scott, Ian B. Hickie

**Affiliations:** 1 Brain and Mind Centre, University of Sydney, Sydney, NSW, Australia; 2 Sunshine Coast Mind and Neuroscience Thompson Institute, University of the Sunshine Coast, Birtinya, Queensland, Australia; 3 St Vincent’s and Mater Clinical School, The University of Notre Dame, Sydney, NSW, Australia; University of Toronto, CANADA

## Abstract

**Background:**

A priority for health services is to reduce self-harm in young people. Predicting self-harm is challenging due to their rarity and complexity, however this does not preclude the utility of prediction models to improve decision-making regarding a service response in terms of more detailed assessments and/or intervention. The aim of this study was to predict self-harm within six-months after initial presentation.

**Method:**

The study included 1962 young people (12–30 years) presenting to youth mental health services in Australia. Six machine learning algorithms were trained and tested with ten repeats of ten-fold cross-validation. The net benefit of these models were evaluated using decision curve analysis.

**Results:**

Out of 1962 young people, 320 (16%) engaged in self-harm in the six months after first assessment and 1642 (84%) did not. The top 25% of young people as ranked by mean predicted probability accounted for 51.6% - 56.2% of all who engaged in self-harm. By the top 50%, this increased to 82.1%-84.4%. Models demonstrated fair overall prediction (AUROCs; 0.744–0.755) and calibration which indicates that predicted probabilities were close to the true probabilities (brier scores; 0.185–0.196). The net benefit of these models were positive and superior to the ‘treat everyone’ strategy. The strongest predictors were (in ranked order); a history of self-harm, age, social and occupational functioning, sex, bipolar disorder, psychosis-like experiences, treatment with antipsychotics, and a history of suicide ideation.

**Conclusion:**

Prediction models for self-harm may have utility to identify a large sub population who would benefit from further assessment and targeted (low intensity) interventions. Such models could enhance health service approaches to identify and reduce self-harm, a considerable source of distress, morbidity, ongoing health care utilisation and mortality.

## Introduction

The ability to predict future death by suicide is still not much better than chance [1–3]. Yet, self-harm, which includes any intentional acts to self-injure irrespective of motivational intent behind these actions (i.e. suicide attempts and non-suicidal self-injury) [4–6], are the source of ongoing distress, morbidity and health care utilisation [7, 8]. Young people presenting to health services represent a group particularly at risk given the early age of onset of self-harm and their strong association with mental disorders [5, 9–11]. Consequently, a focus on predicting those at greatest risk of self-harm (rather than simply death by suicide) is an important goal for services.

Although factors such as suicidal thoughts, depression and alcohol misuse are consistently associated with future self-harm [12, 13], there is still significant doubt about the actual clinical utility of these factors for individual risk predictions [3, 14]. The problem extends to other biological [15] or clinical [16, 17] risk factors which have similarly weak predictive value. Self-harm is likely to be driven by the complex interplay between a broad range of social, biological, psychological, and contextual factors rather than any one or simple set of factors [18]. Further, the influence these factors have on self-harm is probably dynamic over time. The use of modern data science methods may help us overcome some of these challenges by considering the high-dimensional interactions between a large set of variables [19]. These methods attempt to embrace the complexity of the problem, which may be better suited to yield findings that reflect the real-world experiences of clinicians who are asked to solve these complex classification problems every day. Such approaches have been used to predict suicide and self-harm in a range of hospital or outpatient settings [20–24].

Inevitably, the low prevalence of self-harm in many of these populations means there are major statistical limitations [25]. While, some have argued that this limits the clinical utility of such models [26], others have suggested that positive predictive value (PPV) alone is not the criterion for evaluating the utility of these models [27]. The intended use of a model is important to evaluate its clinical utility on balance of its benefits and harms [28]. Specifically, is the model being used to determine who should be admitted to hospital (i.e. a highly invasive and costly intervention), or who should be recommended a more detailed assessment (i.e. a low cost, non-invasive intervention). For this, decision curve analysis (a measure of net benefit) can be used to evaluate prediction models in a way that takes into account the relative benefit of an intervention for a true-positive case versus the cost of an intervention for a false-positive case [29]. Rejecting these tools based on their low PPVs implicitly assumes a high decision threshold, yet we know that in other areas of medicine, low thresholds for intervention are common when there is a lower cost to intervention (e.g. the prescription of statins) [28, 30]. Consideration of the cost and benefits of intervention thresholds are required to advance the development of useful clinical decision-making tools.

In this study we utilise a large clinical cohort of young people presenting to for youth mental health care to determine whether demographic and clinical characteristics at first assessment could be used to predict self-harm within the next six months. The goal here is to apply machine learning methods to evaluate the net benefit of these prediction models, and to identify factors that are consistently associated with self-harm in this clinical population. This study focuses specifically on clinical relevance and service allocation for those entering clinical services so a shorter time frame was deemed to be a suitable follow-up period to maximise the implications for immediate clinical decision-making, and the broader definition of self-harm (i.e. suicide attempts and non-suicidal self-injury) was used to capture all harmful behaviours that would initiate service response in terms of more detailed assessment and/or intervention.

## Material and methods

The study was approved by the University of Sydney Human Research Ethics Committee (2008/5453, 2012/1626) and participants gave written informed consent.

### Participants

Participants are drawn from a cohort of 6743 individuals aged 12–30 who presented to the Brain and Mind Centre’s youth mental health clinics in Sydney and recruited to a research register between June 2008 and July 2018 [31]. These clinics include primary care services (i.e. *headspace* [32, 33]) as well as more specialised mental health services. Young people may have been self-referred, referred via a family member or friend, or the community (e.g. general practitioner) [33]. All participants received clinician-based case management and psychological, social, and/or medical interventions as part of standard care.

### Eligibility criteria

As of December 2019, longitudinal data were available for N = 2901 participants. Of these 2901 participants, the inclusion criteria for potential participants to be included in this specific study were: (i) aged 12 to 30 years at the time of initial visit; and (ii) a follow-up visit within six months of initial visit. Application of these criteria reduced the sample to 1962 individuals.

### Data collection

Data were extracted from clinical files, and code inputs according to proforma (i.e. standardised form) [31, 34]. The proforma records information at predetermined time points. The first available clinical assessment at the service is taken as the baseline time point for each participant and the date of this assessment is used to determine each of the follow up time points. If there is no clinical information available for any time point (i.e. the participant did not attend the service during that time) then that entry is left missing. All clinical notes from the preceding time points, up to and including the current time point are used to inform and complete the current pro forma entry.

The proforma was used to record specific illness course characteristics. More detailed descriptions about the proforma, including the interrater reliability, are reported in the supplement and cohort paper [31]. The measures used here include (see S1 Data); demographics, social and occupational functioning (including, the Social and Occupational Functioning Assessment Scale (SOFAS; [35]), and Not in Education, Employment or Training (NEET) as a measure of participation and engagement with education or work), mental disorder diagnoses, clinical stage, at-risk mental states, self-harm, suicidal thoughts and behaviours, physical health comorbidities, personal mental illness history, and treatment utilisation.

The presence of suicidal ideation, suicide attempts, and non-suicidal self-injury is recorded. A suicide attempt is recorded when a young person has undergone steps to take their own life. If an individual harmed themselves via cutting, hitting themselves, burning themselves, or scratching with the intention to self-harm only and not to take their life, then this is included as non-suicidal self-injury and not a suicide attempt. If a suicide attempt occurs, it is also recorded whether the attempt resulted in hospitalisation or presentation to a hospital emergency department. For the present study, the ‘suicide attempt’ and ‘non-suicidal self-injury’ variables were combined under the broad definition of self-harm and used as the primary outcome measure. This is consistent with current conceptualisations of non-suicidal self-injury and suicide attempts which recognise that the separation of non-suicidal self-injury and suicide attempts on the basis of apparent motivations (i.e. suicide intent) may be unwarranted. The dimensional nature of suicidal intent phenomena means that accurate characterisation of these behaviours is challenging, and so national guidelines tend to broadly focus on self-harm [36, 37]. For this reason, we use this broader definition of self-harm so that we capture harmful behaviours that are likely to be the drivers of service response in terms of assessment and/or intervention.

### Statistical analysis

The assembled dataset consisted of 37 basic demographic and clinical variables to predict whether or not the patient will report self-harm in any follow-up visit within six months of baseline. Categorical predictor variables with a small number of observations were removed [38]. Here, we set the threshold for variables with uncommon observations at 25. All variables for all patients were complete except for “physical health problems–other” where any missing observations (N = 107) were imputed as absent.

We followed the analysis approach described in previously published work [39]. Briefly, models were trained and tested with ten repeats of ten-fold cross-validation. At each fold of the cross-validation, the training set was balanced with three approaches. Firstly, the number of patients in the minority class (cases of self-harm) was doubled using SMOTE [40] to synthetically generate cases. Secondly, borderline samples identified as ‘Tomek links’, which are pairs of similar samples from different classes [41], were removed. Finally, we randomly under-sampled the majority class to balance with the minority class. The test set remained unaltered to assess the models’ performance on the real-world distribution of self-harm.

Following the “No Free Lunch” theorem [42], a number of algorithms were implemented to build predictive models. These algorithms were chosen based on a number of reasons: their popularity in the literature [43], ability to perform both predictive modelling and variable selection [44], has been utilised in other work on self-harm and suicidality [24, 45], can handle both continuous and categorical variables, and some of the chosen algorithms can model non-linear relationships between predictor variables. The algorithms were (i) Area Under the Curve Random Forests (AUCRF) [46]; (ii) Boruta [47]; (iii) Lasso regression [48]; (iv) Elastic-net regression [49]; and (v) Bayesian Additive Regression Trees (BART) [50] and (vi) Logistic regression. These algorithms, aside from logistic regression, are described in more detail in [39].

The variable selection approach differed for each algorithm. For AUCRF, selected variables are those in the Random Forest model with the highest AUROC. For Boruta, the selected variables must have significantly better importance scores than their permutated form. For LASSO and Elastic-net, the variable must have a non-zero coefficient. For BART, the variable’s inclusion proportion must be greater than a local threshold calculated from the permutation null distribution. For logistic regression, variables were deemed to be selected if the p-value for the variable’s coefficient is ≤0.05.

### Model performance

A range of metrics was used to assess different aspects of model performance: AUROC, the Area under the Precision-Recall Curve (AUPRC) [51], Brier scores [52], sensitivity, specificity, positive predictive value (PPV), negative predictive value (NPV) and net benefit. An AUROC closer to 1 represents a perfect model whereas an AUROC closer to 0.5 represents a weak model. The AUPRC is a measure for imbalanced outcome variables that evaluates between the predicted and true positives [51]. Brier score is a proper scoring function which captures the mean squared error between probabilistic predictions and true outcomes. As such, Brier scores range from 0 to 1 with scores closer to 0 indicating more correct and calibrated predictions. Sensitivity, specificity, PPV and NPV measurements were obtained by dichotomising probabilistic predictions with a cut-off at 0.5. Decision curve analysis [29] uses the net benefit metric as a means to assess the costs and benefits of treatment compared different treatment strategies. The net benefit is calculated using a model’s sensitivity and specificity across different probability thresholds and the outcome’s prevalence in the population. In decision curve analysis, the model with the highest utility is the optimal strategy [53].

Analyses were performed using R [54] version 3.6.2 using packages randomForest [55], Boruta [47], AUCRF [46], bartMachine [56], glmnet [57], caret [58], cluster [59], dplyr [60], ggplot2 [61] and rmda [62].

## Results

A total of 1962 young people were eligible for these analyses with a mean age of 18.36 years (SD = 39.7) and 60% were female. Out of 1962 young people, 320 (16%) engaged in self-harm in the six months after first assessment and 1642 (84%) did not. Of those who did not engage in self-harm, 274 (17%) had suicidal thoughts in the six months after the first assessment. The baseline characteristics of these young people are described in Table 1.

**Table 1:** Baseline demographic and clinical characteristics of variables used for prediction.

	Overall	No	Yes	P-value
**n**	1962	1642	320	
**Sex = Male (%)**	778 (39.7)	702 (42.8)	76 (23.8)	**<0.001**
**Age (mean (SD))**	18.36 (3.57)	18.52 (3.64)	17.52 (3.11)	**<0.001**
**History of self-harm = Yes (%)**	883 (45.0)	616 (37.5)	267 (83.4)	**<0.001**
**History of suicide ideation = Yes (%)**	953 (48.6)	731 (44.5)	222 (69.4)	**<0.001**
**SOFAS (mean (SD))**	62.43 (9.02)	62.75 (9.06)	60.76 (8.62)	**<0.001**
**NEET = Yes (%)**	307 (15.6)	264 (16.1)	43 (13.4)	0.269
**Clinical stage (%)**				**<0.001**
Stage 1a	604 (30.8)	541 (32.9)	63 (19.7)	
Stage 1b	1207 (61.5)	973 (59.3)	234 (73.1)	
Stage 2 and above	151 (7.7)	128 (7.8)	23 (7.2)	
**Depression (%)**				**<0.001**
No	598 (30.5)	538 (32.8)	60 (18.8)	
Full-threshold	633 (32.3)	494 (30.1)	139 (43.4)	
Sub-threshold	731 (37.3)	610 (37.1)	121 (37.8)	
**Anxiety (%)**				0.149
No	587 (29.9)	483 (29.4)	104 (32.5)	
Full-threshold	624 (31.8)	515 (31.4)	109 (34.1)	
Sub-threshold	751 (38.3)	644 (39.2)	107 (33.4)	
**Bipolar disorder (%)**				0.957
No	1780 (90.7)	1490 (90.7)	290 (90.6)	
Full-threshold	71 (3.6)	60 (3.7)	11 (3.4)	
Sub-threshold	111 (5.7)	92 (5.6)	19 (5.9)	
**Psychosis (%)**				0.204
No	1862 (94.9)	1553 (94.6)	309 (96.6)	
Full-threshold	44 (2.2)	41 (2.5)	3 (0.9)	
Sub-threshold	56 (2.9)	48 (2.9)	8 (2.5)	
**Psychosis-like experiences = Yes (%)**	356 (18.1)	282 (17.2)	74 (23.1)	0.014
**Mania-like experiences = Yes (%)**	298 (15.2)	240 (14.6)	58 (18.1)	0.13
**Circadian disturbance = Yes (%)**	294 (15.0)	244 (14.9)	50 (15.6)	0.791
**History of hospitalisation = Yes (%)**	346 (17.6)	265 (16.1)	81 (25.3)	**<0.001**
**Any childhood disorders = No (%)**	1691 (86.2)	1404 (85.5)	287 (89.7)	0.058
**Childhood autism spectrum = Yes (%)**	56 (2.9)	52 (3.2)	4 (1.2)	0.089
**Childhood ADHD = Yes (%)**	104 (5.3)	94 (5.7)	10 (3.1)	0.078
**Childhood generalised anxiety = Yes (%)**	41 (2.1)	32 (1.9)	9 (2.8)	0.439
**Childhood depression = Yes (%)**	30 (1.5)	24 (1.5)	6 (1.9)	0.762
**Family history—bipolar = Yes (%)**	154 (7.8)	135 (8.2)	19 (5.9)	0.202
**Family history—psychosis = Yes (%)**	82 (4.2)	69 (4.2)	13 (4.1)	1
**Family history—suicide = Yes (%)**	35 (1.8)	28 (1.7)	7 (2.2)	0.715
**Family history—depression = Yes (%)**	653 (33.3)	542 (33.0)	111 (34.7)	0.604
**Family history—anxiety = Yes (%)**	302 (15.4)	248 (15.1)	54 (16.9)	0.472
**Family history—alcohol = Yes (%)**	171 (8.7)	134 (8.2)	37 (11.6)	0.062
**Family history—substance misuse = Yes (%)**	140 (7.1)	118 (7.2)	22 (6.9)	0.937
**Any physical health problems = No (%)**	1598 (81.4)	1332 (81.1)	266 (83.1)	0.444
**Endocrine problem = Yes (%)**	64 (3.3)	50 (3.0)	14 (4.4)	0.292
**Metabolic problem = Yes (%)**	35 (1.8)	31 (1.9)	4 (1.2)	0.577
**Neurological problem = Yes (%)**	55 (2.8)	49 (3.0)	6 (1.9)	0.36
**Physical health problems—Other = Yes (%)**	257 (13.1)	222 (13.5)	35 (10.9)	0.245
**Treatment antidepressants = Yes (%)**	737 (37.6)	606 (36.9)	131 (40.9)	0.194
**Treatment antipsychotics = Yes (%)**	239 (12.2)	195 (11.9)	44 (13.8)	0.398
**Treatment mood stabilisers = Yes (%)**	124 (6.3)	102 (6.2)	22 (6.9)	0.749
**Treatment stimulants = Yes (%)**	166 (8.5)	152 (9.3)	14 (4.4)	0.006
**Treatment psychological therapy = Yes (%)**	1101 (56.1)	927 (56.5)	174 (54.4)	0.532

**Note.** Columns present overall statistics and then statistics split by whether or not patients exhibited self-harm within 6 months of presentation (No vs Yes). P-value is reported for univariate comparisons between downstream self-harm. For categorical variables, the Chi-square test is performed and for continuous variables, the t-test is performed. Bolded text indicates best significant results at P<0.001.

### Model performance

All six algorithms produced predictive models with similar performance across all metrics in the test datasets (Table 2). One-hundred models were produced for each algorithm (600 in total) and on average, Boruta random forest models had the highest AUPRC (Mean: 0.346, SD: 0.056), PPV (Mean: 0.321, SD: 0.035) and specificity (Mean: 0.722, SD: 0.037), and also the lowest Brier scores (Mean: 0.185, SD: 0.014). Lasso regression models had the highest mean NPV (Mean: 0.934, SD: 0.018) and sensitivity (Mean: 0.752, SD: 0.078), and BART had the highest mean AUROC (Mean: 0.755, SD: 0.039).

**Table 2:** Machine learning model performance.

Metric	AUCRF	Bart	Boruta	Elastic-net	LASSO	Logit
**AUROC**	0.749 (0.037)	**0.755 (0.039)**	0.749 (0.035)	0.749 (0.04)	0.749 (0.04)	0.744 (0.041)
**AUPRC**	0.342 (0.058)	0.329 (0.055)	**0.346 (0.056)**	0.32 (0.051)	0.32 (0.051)	0.316 (0.052)
**Brier Score**	0.19 (0.025)	0.188 (0.016)	**0.185 (0.014)**	0.193 (0.014)	0.193 (0.015)	0.196 (0.016)
**Sensitivity**	0.684 (0.083)	0.733 (0.076)	0.67 (0.079)	0.735 (0.071)	**0.752 (0.078)**	0.7 (0.083)
**Specificity**	0.715 (0.041)	0.695 (0.041)	**0.722 (0.037)**	0.686 (0.043)	0.678 (0.043)	0.704 (0.037)
**PPV**	0.321 (0.032)	0.321 (0.033)	0.321 (0.035)	0.315 (0.03)	0.314 (0.03)	**0.316 (0.034)**
**NPV**	0.921 (0.018)	0.931 (0.018)	**0.919 (0.018)**	0.93 (0.017)	0.934 (0.018)	0.924 (0.02)

**Note.** The means and standard deviations of model performance on test sets across the ten repeats of 10-fold cross-validation. AUROC = Area under the receiver-operator curve, AUPRC = Area under the precision-recall curve. Sensitivity, Specificity, Positive Predictive Value (PPV) and Negative Predictive Value (NPV) were calculated using a probability threshold of 0.5. Bolded text indicates best performing model for each metric.

**Fig 1:**
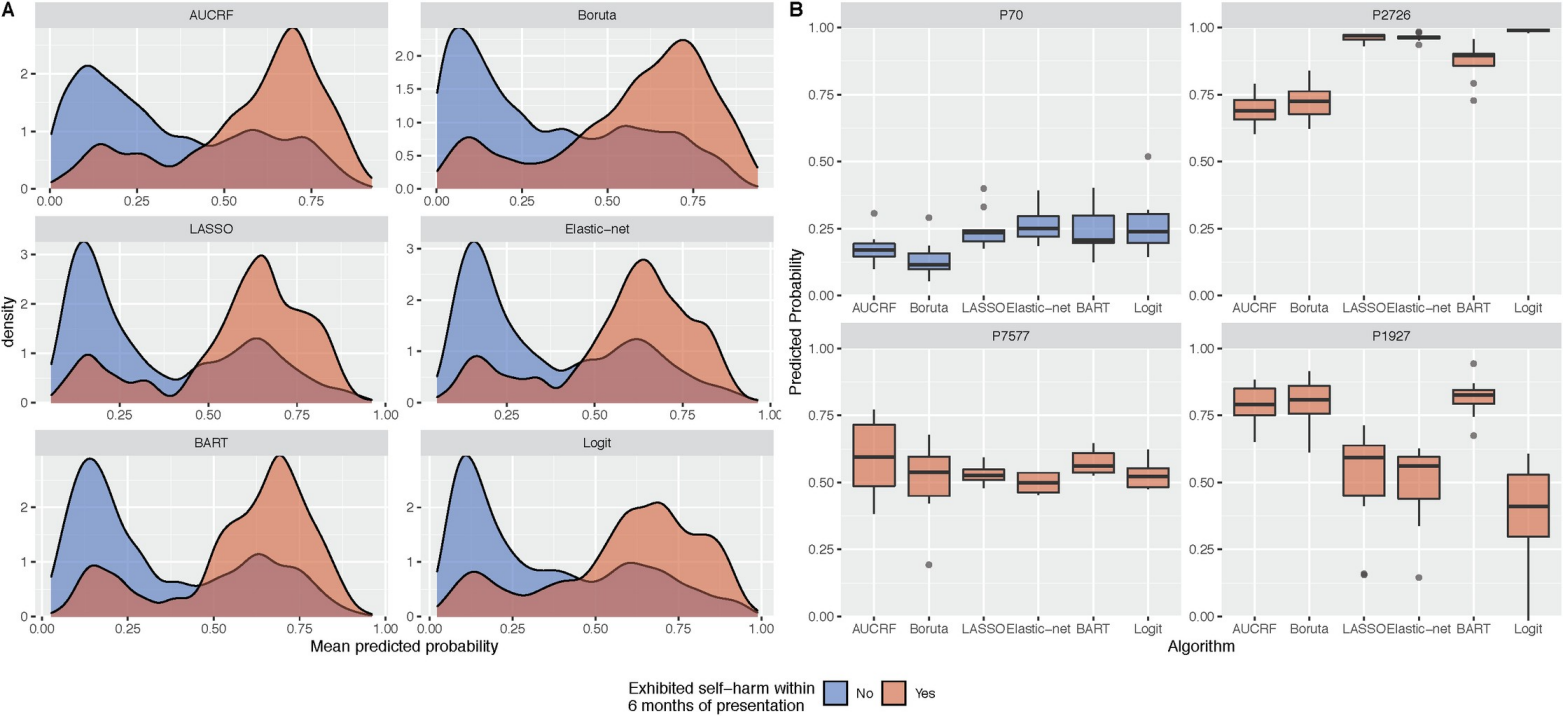
Predicted probabilities of self-harm across the six machine learning algorithms. Panel A presents the distribution of mean predicted probabilities. The mean predicted probabilities for each patient are averaged across ten repeats of 10-fold cross-validation. Panel B presents the uncertainty in predicted probabilities for a selection of patients. Boxplots show the predicted probabilities across the five algorithms and repeated cross-validation. The predictions for person ID 70 would suggest that the person is highly unlikely to engage in self-harm, in contrast to person ID 2726. In some instances, such as person ID 7577, models could not distinguish whether a person would or would not engage in self-harm as the predicted probabilities are close to 0.5. There are instances where the models will conflict in their predictions (e.g. person ID 1927).

The mean predicted probabilities for each patient, averaged across the ten repeats of cross-validation, skewed towards zero for the majority for patients who did engage in self-harm and, for those who did, it skewed towards one (Fig 1A). The tails of these distributions suggest that there are still a number of patients whom the models incorrectly classified. The top 25% of patients as ranked by this mean predicted probability accounted for 51.6% - 56.2% of all patients who exhibited self-harm. By the top 50% of ranked patients, this increased to 82.1% - 84.4%. The variance in predicted probabilities for each person across different algorithms and repeats of cross-validation can inform on the uncertainty of the model predictions (Fig 1B).

**Fig 2:**
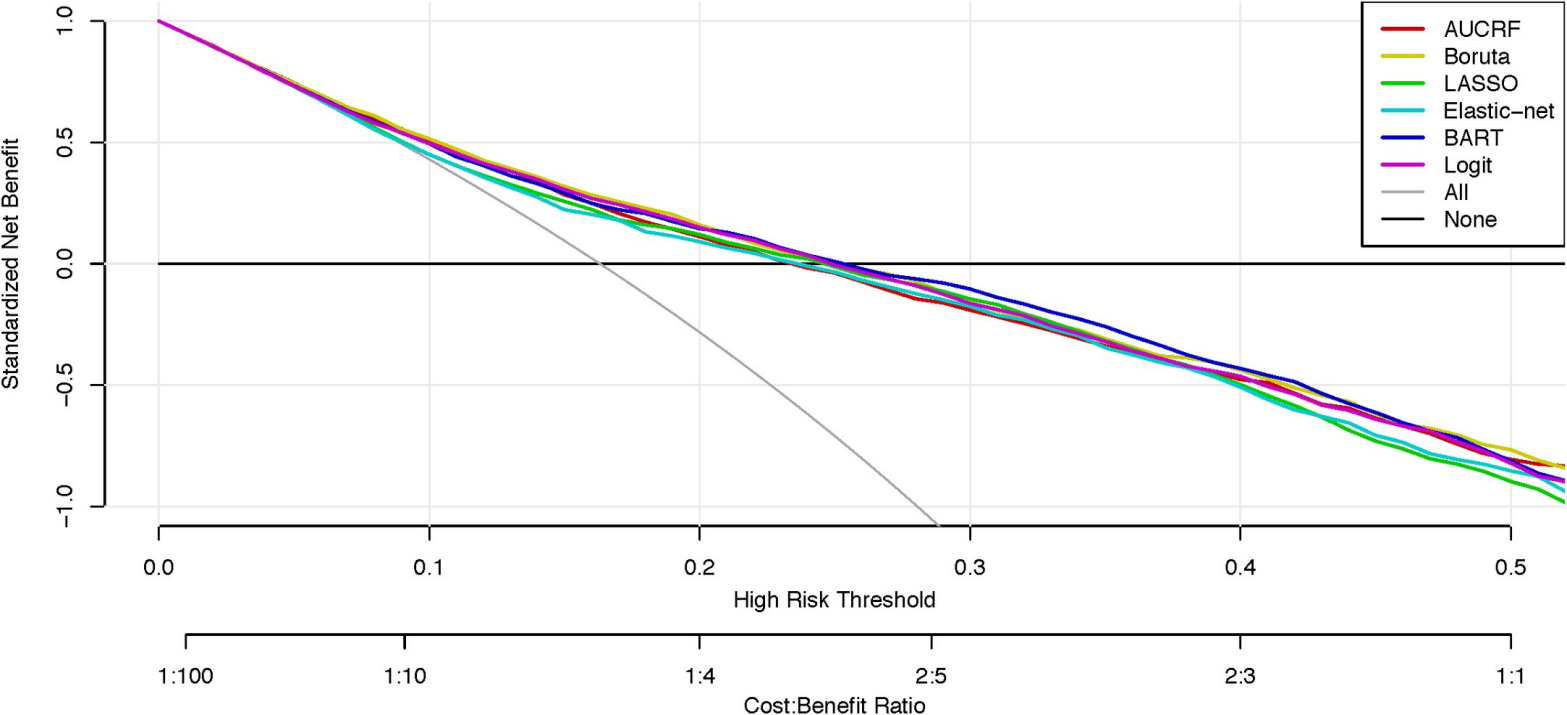
Decision curve analysis of machine learning models predicting self-harm. Net curves are plotted across a range of probability thresholds for self-harm. The grey line plots the assumption that all people will engage in self-harm (i.e. ‘treat everybody’), whereas the black line assumes that no one will engage in self-harm (i.e. ‘treat no one’). The six coloured lines plot the net benefit of using machine learning models to identify who will engage in self-harm.

The decision curve was produced also using the mean predicted probabilities and contrasted against strategies to ‘treat everyone’ and ‘treat no one’ (Fig 2). All six models were superior to the ‘treat everyone’ strategy and the net benefit of these models were positive for thresholds between 0.09 and 0.26. The curves for all six algorithms remain very close with BART being the marginally superior model.

### Key predictors

**Fig 3:**
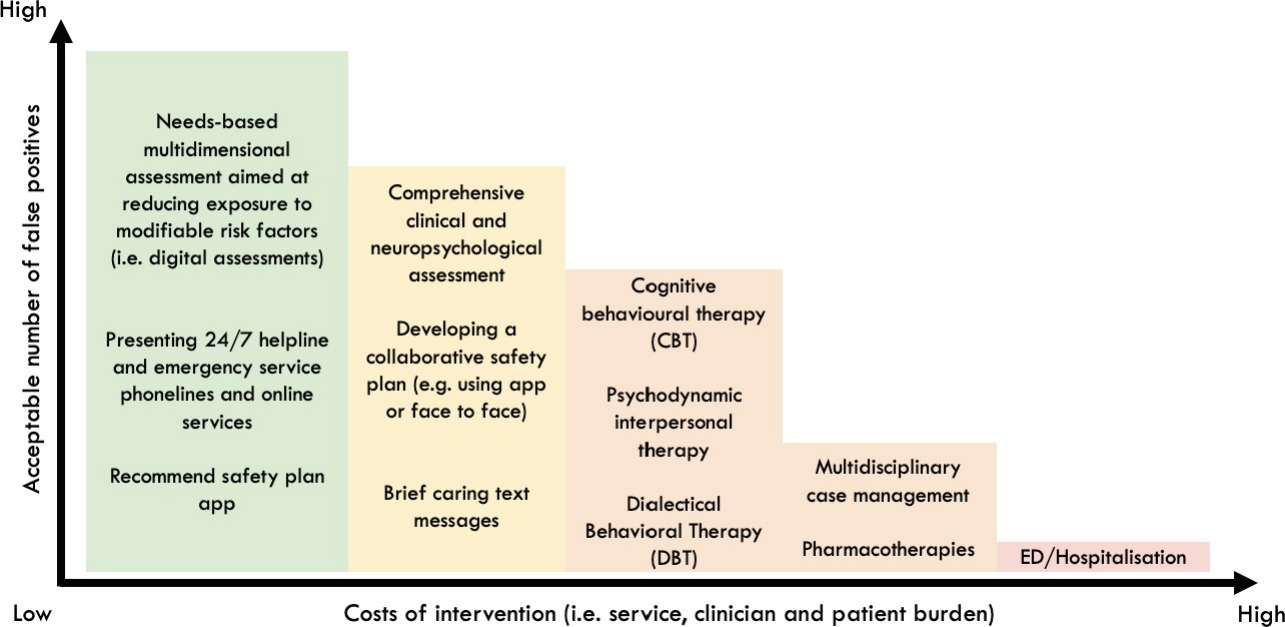
Hypothetical graph that weighs up the net benefit of different interventions in youth mental health settings. This hypothetical graph weighs up the costs of intervention (i.e. individual and clinician burden) against the number of people we are willing to treat in order to prevent future self-harm. As the costs of intervention increase, naturally the acceptable number of false positives reduce because intervention is likely to result in greater costs than benefits (resulting in a negative net benefit). However, when the costs of intervention are low, a higher number of false positives are acceptable to successfully prevent one case of self-harm (resulting in positive net benefit).

Variable importance for predicting self-harm are presented in Figs 2 and 3 of the online supplement. There were 7 predictors that were selected in at least 80% of the models (480/600). In rank order, these predictors are: (1) a history of self-harm; (2) age; (3) SOFAS score; (4) sex; (5) bipolar disorder; (6) psychosis-like experiences; (7) treatment with antipsychotics.

## Discussion

A major priority for mental health services is to prevent self-harm, which is a considerable source of distress, morbidity, ongoing health care utilisation and mortality, particularly in youth. This study evaluates the potential utility of machine learning as a tool that can improve clinical decision-making. First, in a cohort of young people presenting to youth mental health services, the machine learning models here demonstrated fair overall prediction (AUROCs between 0.744 and 0.755) and were well calibrated which indicates that predicted probabilities were close to the true probabilities (brier scores between 0.185 and 0.196). Second, the decision curve analysis indicates that there was a net benefit of these models over a ‘treat everybody’ approach, suggesting the potential to allocate targeted assessments and interventions in addition to those broad health service strategies. Finally, we identified seven basic factors that were among the strongest predictors and demonstrate the relative importance of these characteristics to identify those who may be at risk for self-harm in young people with emerging mental disorders.

Most prediction studies for self-harm have focussed on adults presenting to hospital or emergency departments, other high-risk populations, or focussed exclusively on suicide attempts. The application of this approach to a younger cohort with greater clinical heterogeneity and who may not have a prior history of self-harm or serious mental disorders is novel. Developing such prediction models for different populations and settings is critical given the complexity of self-harm [3]. We report classification performance metrics that are comparable to many previous prediction models [20–24], and comparable or better than most clinical instruments used in high-risk populations [63]. This level of performance was achieved using only basic demographic and clinical factors, common to many intake assessments and not as rich as more comprehensive digital assessments now available [64]. The clinical context is an important consideration given that these young people are typically early in the course of illness or never sought help before [65], and almost one in five (17%) cases were new onset self-harm.

Clinicians are asked to make decisions every day about who requires further assessment and what type of treatment is most likely to be appropriate and effective for an individual. The lack of useful markers of illness means that these decisions are generally based on broad clinical guidelines, risk assessment tools and clinical intuition, each of which have major limitations. Most international guidelines recommend a needs-based assessment in high-risk settings [37], yet carrying out such assessments can be time and resource intensive, lead to the use of informal triage rules [66, 67], or rely on unvalidated locally-developed proformas [67]. Furthermore, there are major limitations to relying on clinical judgements for a range of outcomes, including future self-harm [68, 69]. Together, this reiterates the challenges health services and clinicians face when trying to prevent self-harm. There is a need for innovative health service approaches that can improve the consistency, effectiveness and safety of clinical decision-making [70].

The decision curve analysis can be used to stimulate discussion within services about the cost-benefits of different interventions across a range of risk thresholds. For thresholds between 0.09 and 0.26 all models presented here have a net benefit that is higher than a ‘treat everyone' approach. So, at low thresholds such models may have utility for allocating low intensity interventions in a way that optimises the cost-benefits. In practice, everyone presenting for youth mental health care could receive a needs-based digital assessment that includes the assessment of suicidal thoughts and self-harm (‘treat everyone’ approach) [64, 71]. This health service strategy reflects international guidelines and approaches to reduce risk (e.g. zero suicide) [70, 72, 73]. For those above a low threshold (~0.20), a further assessment and low intensity interventions could be recommended [74, 75]. This next level of assessment or intervention may be viewed as inappropriate or unfeasible to be provided to everyone but recommending it to a large subpopulation is acceptable to prevent one case of self-harm (Fig 3). These additional resources may be even more cost effective when considering that those identified at risk for self-harm tend to also be at risk for a range of negative mental health outcomes [7, 76].

A better understanding of key model predictors may be helpful to inform clinical decision making for reducing self-harm [37]. While, caution should be taken when interpreting variable rankings [77], many of the variables were highly intuitive and clinically informative. Consistent with previous findings, a history of self-harm predicted future self-harm in youth, even when considered among a range of other clinical factors [3, 14]. Interestingly though, social and occupational functioning was the third highest predictor, ranked higher than all other clinical factors (e.g diagnoses, previous hospitalisation). Maladaptive social and occupational factors have been associated with self-harm in youth and tends to include; adverse or absent social relationships [78], and poor educational or employment participation [34]. This work suggests that poor social and occupational functioning may be a critical target for intervention to reduce self-harm for some.

The association between self-harm and mental or substance use disorders has been widely reported in youth [5, 79, 80]. Mental disorder diagnoses were selected in our models to predict future self-harm, however the frequency of which these variables were selected was less than expected relative to other variables in our study. Bipolar disorder and psychosis-like experiences were among the strongest predictors, yet while depression was less important, they were still selected in over 70% of models.

### Limitations and future directions

These findings should be considered in the context of some limitations. First, the sample size used here is relatively small and there was a major class imbalance for the main outcome. Second, we only considered a limited set of categorical variables. There are a range of additional social and contextual factors not considered here which may have influenced the results. Third, we only use baseline variables to predict future self-harm, however these models could benefit from time varying predictions [81]. Though, the use of baseline variables only does serve to replicate real-world clinical decision making after an initial presentation to a service. We used a limited set of machine learning algorithms that provided the opportunity for variable selection. Future studies should consider the utility of these models compared to clinician ratings, or a combination of these to make more informed decisions.

Females made up nearly three quarters of those who exhibited self-harm. Evidence suggests that low-to-moderate self-harm (e.g. superficial cutting etc.) tend to be more common among females, while males tend to engage in methods of self-harm that are more severe and likely to result in suicide (e.g., hanging, firearms) [82]. In light of this, the self-harm identified by this study are most likely low-to-moderate in severity. A more comprehensive understanding of self-harm methods is a matter for future studies.

The costs and benefits implicitly modelled in this work assume these are uniform for the entire population. While this may be true, a more detailed evaluation of costs and benefits for subgroups within this population may be required to accurately model these to inform decision making. Similarly, further research may also benefit from predicting these outcomes among subpopulations within the service whereby self-harm are particularly common (i.e. borderline or complex cases) [83]. These results may provide the opportunity for increase personalisation of interventions, improvements in prediction performance and greater cost-benefit ratios.

## Conclusion

The present work supports the view that data driven, and machine learning methods have the potential to advance clinical decision making for self-harm [30]. This study demonstrates the potential clinical utility of prediction models to identify a large sub population who may benefit from targeted (low intensity) interventions in addition to the broad health service prevention strategies. Enhancing how health services identify and respond to self-harm is a critical priority, not simply because of the risk they confer for future suicide, but due to the significant distress, morbidity and ongoing health care utilisation associated with self-harm.

## Supporting information

S1 Data (CSV)

S1 File (DOCX)
